# Chloroform in Obstetrics

**Published:** 1854-05

**Authors:** 


*Chloroform in Obstetrics.*

We were surprised to see that in 180 cases of midwifery, in which chloroform was used, Dr. Barwell did not meet with a single case in which its use promoted the dilation of the os uteri and perinæum, and increased the secretion of the vagina. The experience of M. Caseaux confirms this observation, and reports a case in which very extensive laceration of the perinæum occurred while the patient was under the influence of chloform. But most who have had any considerable experience in its use have come tc a different opinion. In our own practice we have several times seen the soft parts very rapidly relax after the inhalation of chloroform where the rigidity of these parts had previously constituted the great obstacle to the immediate termination of the labor. In more than one case we have seen it completed without assistance where the chloroform was used as a preparation for forceps delivery.—*American Medical Monthly.*

